# Effects of Dietary α-Linolenic Acid Treatment and the Efficiency of Its Conversion to Eicosapentaenoic and Docosahexaenoic Acids in Obesity and Related Diseases

**DOI:** 10.3390/molecules27144471

**Published:** 2022-07-13

**Authors:** Marija Takic, Biljana Pokimica, Gordana Petrovic-Oggiano, Tamara Popovic

**Affiliations:** Group for Nutrition and Metabolism, Institute for Medical Research, National Institute of Republic of Serbia, University of Belgrade, 11000 Belgrade, Serbia; biljana.pokimica@imi.bg.ac.rs (B.P.); gordana.ogiano@imi.bg.ac.rs (G.P.-O.); poptam@imi.bg.ac.rs (T.P.)

**Keywords:** ALA, obesity, omega-3, flax seed, chia, walnut

## Abstract

The essential fatty acid alpha-linolenic acid (ALA) is present in high amounts in oils such as flaxseed, soy, hemp, rapeseed, chia, and perilla, while stearidonic acid is abundant in echium oil. ALA is metabolized to eicosapentaenoic acid (EPA) and docosahexaenoic acid (DHA) by desaturases and elongases in humans. The conversion of ALA to EPA and DHA is limited, and these long-chain n−3 polyunsaturated fatty acids (PUFAs) are mainly provided from dietary sources (fish and seafood). This review provides an overview of studies that explored the effects of dietary supplementation with ALA in obesity and related diseases. The obesity-associated changes of desaturase and elongase activities are summarized, as they could influence the metabolic conversion of ALA. Generally, supplementation with ALA or ALA-rich oils leads to an increase in EPA levels and has no effect on DHA or omega-3 index. According to the literature data, stearidonic acid could enhance conversion of ALA to long-chain n−3 PUFA in obesity. Recent studies confirm that EPA and DHA intake should be considered as a primary dietary treatment strategy for improving the omega-3 index in obesity and related diseases.

## 1. Introduction

Alpha-linolenic acid (ALA) is an essential fatty acid, and it is classified as an n−3 polyunsaturated fatty acid (PUFA) [1]. Some plant foods contain considerable amounts of ALA such as flaxseed, perilla, chia seeds, walnuts, rapeseed, and soybeans [2]. The mean dietary intake of ALA is generally slightly above the adequate intake recommendation [3,4,5]. An intriguing scientific question is whether ALA exerts its physiological benefits only after its metabolism to the active forms eicosapentaenoic acid (EPA) and docosahexaenoic acid (DHA) or shows unique effects in organisms [6,7,8]. Therefore, it could be of great importance to follow changes in EPA and DHA status during ALA supplementation. Another question that arises is whether EPA+DHA (fish oil) or ALA consumption produces more favorable biological changes in the investigated cardiometabolic parameter. Furthermore, the expansion of current knowledge of ALA’s in vivo mechanisms of action could provide better insight into the potential roles of ALA consumption. 

Obesity is a worldwide health problem with high and increasing prevalence [9]. Many nutrition strategies for the prevention of obesity, as well as its management, focus on the dietary intake of PUFAs [10,11]. Moreover, common obesity-associated features such as dyslipidemia, hypertension, and insulin insensitivity [12,13,14] that lead to the development of metabolic syndrome and increase the risk for the development of cardiovascular and other diseases such as type 2 diabetes and cancer are not found in all obese subjects [15,16]. ALA may play an important role in obesity and related diseases as well as obesity-associated comorbidities [17,18,19], but its effects are generally inconsistent and understudied, so future well-designed controlled clinical trials could be needed. In addition to the listed features associated with obesity, the ectopic accumulation of the fat in the liver is often present, and it can lead to the development of non-alcoholic fatty liver disease (NAFLD) [20]. Thus, modification of lifestyle factors that lead to obesity may be the cornerstones in NAFLD management [21], and dietary intervention using ALA could be a very important scientific topic. Finally, it seems that overweight and obesity *per se* influence fatty acid status in circulation and tissues, affecting desaturase and elongase activities, i.e., endogenous fatty acid metabolism, which has been covered in the systematic review by Fekete et al. [22]. Since the effects on fatty acid profiles of serum or other body compartments during interventional studies with ALA are still not routinely estimated, future investigations of the influence of ALA on PUFA status in circulation and tissues in obesity could be of great importance.

In this context, this review focuses on ALA effects in overweight/obesity. We give a brief overview of current knowledge of the effects of two frequently used natural dietary sources of ALA (flaxseed and walnuts). We explore the available literature data and present an overview of recent studies regarding ALA effects in obesity focusing on in vivo mechanisms of action and effects on features associated with obesity, among which the knowledge of blood pressure and vascular health has been significantly updated and expanded in recent years. The specific aim of this review was to summarize the knowledge of PUFA profiles in obesity and present data on ALA interventions on PUFA status in obesity. Finally, as stearidonic acid increases the desaturation/elongation of PUFAs, current knowledge of its effect on obesity is briefly discussed.

## 2. Literature Search and Study Selection

This narrative review was based on PubMed electronic database search for relevant publications using the following terms: (“linolenic acid” OR “obesity”) AND “obesity” AND (“fatty acid profiles” OR “polyunsaturated fatty acids” OR “flaxseed” OR “walnuts” OR “chia“) to identify the studies on the association between alpha-linolenic dietary intake/status and obesity and specific clinical dietary intervention studies in patients with obesity. We also focused on systematic reviews with meta-analyses. Studies relevant to this topic, conducted on humans and preferably published in the last 10 years in English, were included in the review. The list of references of studies that are relevant to clinical practice was reduced, and the final list of references was approved with the agreement of the authors. 

## 3. Bioconversion of ALA in an Elongase/Desaturase Pathway

**Figure 1:**
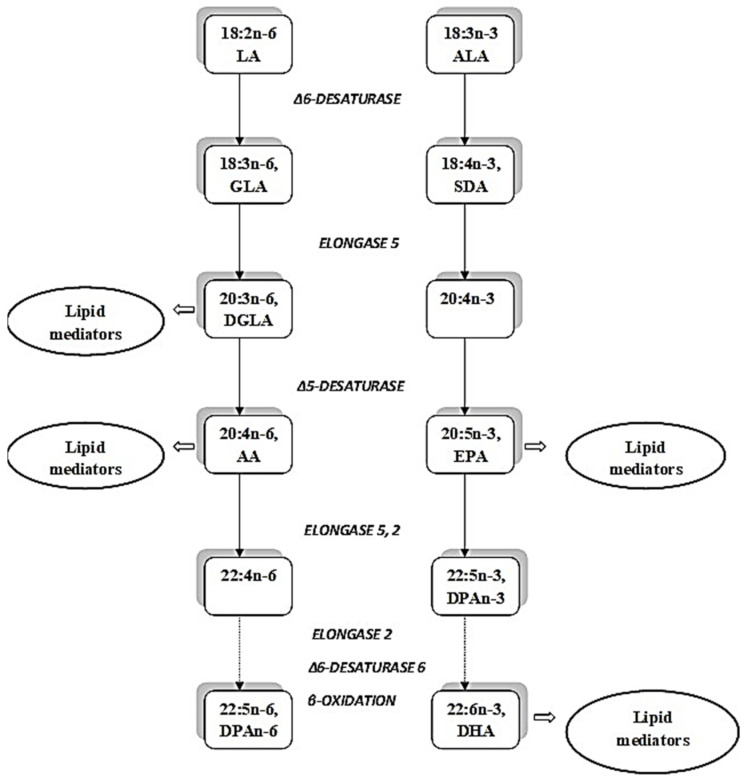
The elongation and desaturation of the essential fatty acids, linoleic acid (LA) and alpha-linolenic acid (ALA). Abbreviations: AA, arachidonic acid; EPA, eicosapentaenoic acid; DPA, docosapentaenoic acid; DHA, docosahexaenoic acid; GLA, γ-linolenic acid; DGLA, di-hommo-γ-linolenic acid; SDA, stearidonic acid.

Linoleic acid (LA) and ALA are essential fatty acids for humans and thus are provided exclusively through dietary consumption [2]. However, both LA and ALA can be bio-converted in the same biochemical pathway by the action of elongation and desaturation enzymes [1] as presented in Figure 1. Thus, the presence of high amounts of LA compared to ALA in diets, a typical characteristic of modern dietary patterns [23] due to competition between LA and ALA for elongation/desaturation enzymes [1,2,24], can result in suppressed metabolic transformation of ALA to eicosapentaenoic acid (EPA) and docosahexaenoic acid (DHA) together with the production of high amounts of arachidonic acid (AA). Di-hommo-γ-linolenic acid (DGLA), AA, EPA, and DHA are precursors for numerous lipid metabolites that exert different physiological effects [1]. Generally, those derived from DGLA, EPA, and DHA are anti-inflammatory, and AA derivates are pro-inflammatory [24]. Therefore, a balanced dietary intake of LA and ALA is a key factor for a nutrition-induced health effect [23].

The gender-specific difference regarding capacity for ALA and LA bioconversion has been revealed [1], and the transformation of ALA to EPA and DHA is more efficient in premenopausal women compared to men [1,25]. At the same time, the efficiency of EPA transformation to DHA is much lower compared to that of ALA to EPA [1,2,25]. In humans, dietary ALA consumption is associated with elevation of ALA and EPA contents in circulation and/or tissues, while there is generally no effect of ALA intake on DHA levels [26,27,28,29,30,31]. LA is present in high amounts in conventional edible vegetable oils, while there are only several plants whose oils are rich in ALA. The main dietary sources of ALA are flaxseed, chia seed, perilla, walnut, rapeseed, and soybean oils [32,33,34,35] as presented in Table 1. The major lipid constituents in conventional edible oils are triglycerides, and up to 10% of fatty acids (FAs) are present in forms of diacylglycerols, monoacylglycerols, and free fatty acids [2,36]. Adequate intake of ALA is 1.1 g/day for women and 1.6g/day for men [37]. The mean intake of ALA in European countries and the US is slightly above the adequate intake [3,5], and doses in interventional trials of about 4 g are easily obtained by consumption of ALA-rich oils or whole seeds. EPA and DHA are not present in high amounts in plants; their primary dietary sources are fish oils and other seafood products, so they are often called marine n−3 PUFAs [2]. The potential beneficial effects of ALA consumption could be attributed to its conversion to active forms of EPA and DHA, but some of the biological effects ALA could also play unique roles, independent of its function as a precursor for long-chain n−3 PUFAs [6,7,8].

## 4. Metabolic Conversion of Polyunsaturated Fatty Acids and Their Status in Obesity and Related Diseases

The excessive accumulation of fat in obesity (especially abdominal fat) has been associated with a high prevalence of hypertension, dyslipidemia (increased triglycerides and decreased high-density lipoproteins (HDLs)), insulin insensitivity, and occurrence of persistent, low-grade chronic inflammation [12,13,14]. The inadequate dietary intake of n−6 and n−3 polyunsaturated fatty acids (PUFAs) may play an important role in the pathophysiology of nutrition-related health problems including those associated with obesity [23]. LA and ALA are essential fatty acids for humans and thus must be provided by the diet. Circulation and tissue PUFA profiles reflect their dietary intake as well as endogenous metabolism to other fatty acids of n−6 and n−3 PUFA families, respectively [1,2,38]. Thus, the FA composition, in addition to the diet, is modified by elongating and desaturation enzyme activities that can be and are usually estimated using product-to-precursor ratios of corresponding fatty acid in most observation and intervention studies [1,39]. The percentage abundance and content of individual n−3 and n−6 PUFAs, especially of those that are not present in high amounts in diets, reflect the LA and ALA metabolic conversion in the desaturase/elongase metabolic pathway [1,40]. According to the available literature data of numerous observational studies, the long-chain polyunsaturated fatty acid (LC-PUFA) status in circulation and tissues could be altered in overweight and obesity [5,12,15,22].

The systematic review and meta-analysis of published data of observation studies regarding PUFA status in overweight/obesity until January 2014 [22] revealed that the level of di-hommo-γ-linolenic acid (DGLA) in phospholipids, cholesterol-esters, and total lipids was significantly increased in overweight or obese subjects compared to controls. The authors observed [5,22] that docosahexadecanoic acid (DHA) content was significantly lower in overweight or obese subjects compared to normal weight controls. In addition, Fekete et al. [22] have noted that the DGLA/linoleic acid ratio (index of delta 6-desaturase activity) was elevated, while the AA/DGLA ratio (index of 5-desaturase activity) decreased in plasma phospholipids. In summary, the results of the meta-analysis of Fekete et al. [22], published in their systematic review, confirm that endogenous PUFA metabolism in obesity could be disturbed, inducing changes in LC-PUFA plasma status in obesity, but primarily regarding n−6 PUFA content. In the last decade, observational studies have generally confirmed that obesity is associated with changes in PUFA metabolism, determining changes in product-to-precursor ratios of corresponding PUFAs and representing indices of desaturase activities [39,40,41,42,43]. In addition to the almost uniform finding that stearoyl-CoA desaturase activity in plasma (mostly reported as SCD16 and SCD18 indices) is increased [39,41,42,44,45,46], numerous studies have reported that delta 6-desaturase activity is positively and delta 5-desaturase inversely related to anthropometric indicators of obesity such as body mass index (BMI), waist circumference, and body fat mass [39,41,42,44]. Some recent observation studies explored the relation between plasma FA profiles and increased body weight including a larger number of participants [39,41,42,43]. The observation study in premenopausal women in Spain exploring the relationships of PUFAs and anthropometric parameters of obesity has revealed that linoleic acid was inversely correlated with BMI and body fat percentage, while direct associations between DGLA and AA levels and these two anthropometric measures were observed [41]. The DGLA serum n−6/n−3 ratio was found to be significantly associated with adiposity measures of women in Mexico [42]. In the largest cross-sectional study to date exploring the connection between serum cholesterol esters’ FA composition and abdominal obesity, the authors found inverse relations between individual PUFAs LA, ALA, and DHA with abdominal obesity [39]. As sex hormones are important regulatory factors in the metabolism of PUFAs, gender-specific differences could exist and should be explored in further studies as data in this area of research are still limited. Contrary to the results of other studies, Yammine et al. [43] found no significant associations between the content of n−6 PUFAs, n−3 PUFAs, or the ratio of n−6/n−3 PUFAs in serum and adiposity parameters (BMI, waist circumference, and percentage of body fat), but divergent relationships between n−3 PUFAs and obesity according to gender were observed. In this study, there was a positive trend for the correlation of BMI and ALA but a negative one for DHA with BMI in men, while γ-linoleic (GLA) and EPA levels were positively associated with this anthropometric parameter in women [41]. Gender-specific differences were noticed in the Alsharari et al. [39] trial as well. The ALA was negatively associated with anthropometric measures in men, while EPA and DHA showed inverse correlations with these measures in women [39]. AA is generally considered to be associated with obesity and metabolic syndrome development. The meta-analysis by Fekete et al. [22] failed to detect significant differences for AA content in any of three plasma lipids fractions, as with the findings of other recent observational studies investigating PUFA status in plasma in obesity and related diseases [39,41,42], while in several studies, positive relationships between the AA level and anthropometric parameters were confirmed [44,46]. Since plasma fatty acid profiles reflect the short-term dietary intake of FAs in the last few days or weeks [47], FA profiles in other body lipid compartments could be better biomarkers for exploring the contribution of PUFAs to overweight, obesity, metabolic syndrome, and other related diseases. Additionally, AA could be utilized for the synthesis of pro-inflammatory eicosanoids [48] as obesity represents a low-grade chronic inflammation state leading to a decrease in this acid concentration. Furthermore, AA and potentially other FAs could be transferred to adipocytes and removed from circulation [49]. At the same time, according to the available literature data [39,43], few reports suggest the association between n−3 PUFA content in serum/plasma and adiposity that has generally believed to exist. Thus, the determination of the FA composition of erythrocytes and tissues (especially of adipose tissue in overweight or obese human subjects) could provide a more informative and reliable biomarker when the association between PUFA profiles and obesity are explored.

Progressive lipid accumulation in obesity is accompanied by alteration of adipose tissue functions [50,51,52], but the mechanisms behind these changes have to be elucidated in future studies. Persistent low-grade inflammation, increased PUFA release in circulation and ectopic fat accumulation are common features accompanying obesity [53]. The characteristic increases in delta-9 desaturase and delta-6 desaturase activities and reduction in delta-5 desaturase found in the plasma in overweight or obese subjects are characteristics of adipose tissue as well [54,55]. However, contrary to reported data for serum/plasma PUFA composition, AA levels in the adipose tissue correlate with increased weight in numerous studies [56,57,58,59]. In the observational study of Savva et al. [56], mean AA, DGLA, and DHA were higher in adipose tissue of overweight and obese children compared to normal weight control, and variance in body mass index (BMI) was better explained (38.2%) by adipose tissue AA content than any other PUFAs. In addition, adipose tissue AA amount was independently associated with abdominal obesity, hypertriglyceridemia, elevated fasting glucose, and high blood pressure in another study [56]. William et al. (2007) reported that subjects with a higher level of AA in adipose tissue showed an increased risk of metabolic syndrome. Greater proportions of palmitoleic and AA in adipose tissue were found in obese twin individuals [58]. Furthermore, according to the literature data, it can be suggested that obesity-induced changes in adipose tissue cells could play a key role in persistent low-grade inflammation [53,60]. So far, studies regarding inflammation in overweight and obese subjects have mostly explored circulation markers of inflammation, although it is well known that PUFAs present in adipose tissue is the precursors for lipid mediators that are involved in inflammation onset [53,60]. Recently, Fisk et al. [61] revealed that greater subcutaneous adipose tissue inflammation in obesity is associated with lower levels of specialized pro-resolving mediators and hydroxyl-DHA metabolites together with altered expression of genes involved in n−3 PUFAs activation, oxylipin synthesis, inflammation, and immune response in adipose tissue. Moreover, dietary treatment with fish oil in the study of Fisk et al. [61] did not induce increased generation of the EPA and DHA metabolites in overweight/obese subjects to the same extent as normal weight individuals. The authors concluded (Fisk 2022) that, based on adipose tissue status, there may be a need for personalized LC n−3 PUFA supplementation in obesity. Furthermore, the results of the study of Fisk et al. [61] emphasize the importance of adipose tissue PUFA status in terms of manifestation of obesity-induced inflammation.

ALA can be metabolized to EPA and DHA by desaturases and elongases. At the same time, an increase in delta 6-desaturase activity together with a reduction in delta 5-desaturase has been found in most studies in plasma [22,41,42,44] and adipose tissue [44,54] as well as in erythrocytes [62] of overweight or obese subjects. It is therefore not easy to predict if the rate of ALA conversion to EPA will be increased or decreased in obesity (Figure 1). As ALA at least partly should exert its potential effects due to conversion to EPA and maybe DHA, human studies estimating the efficiency of conversion in overweight and obese subjects with a comparison to conversion in normal-weight subjects may be needed in order to obtain the needed information for personalized nutritional treatment preparation. Finally, in addition to exploring PUFA status, lipid mediator production in adipose tissue should be monitored to clarify the biological effect of ALA.

## 5. Effects of α-Linolenic Acid Treatment in Obesity, Recent Clinical Trials and Systematic Reviews 

Obesity is a worldwide health problem with increasing prevalence in spite of the fact that it is a largely preventable disease. n−3 PUFAs may play an important role in obesity prevention and management, as well as in the progression of obesity-induced metabolic syndrome [63]. In recent years, it has been noticed that some obese patients are metabolically healthy, insulin sensitive, and have lower fat content and lower intima-media thickness of the carotid artery compared to metabolically "unhealthy" obese ones [15]. On the contrary, in persons living with metabolic syndrome, abdominal obesity has been accompanied by dyslipidemia (high serum triglycerides and low HDL lipoprotein content), impaired glucoregulation, and hypertension leading to an increase in the risk of coronary heart disease, cerebrovascular disease, and all-cause mortality [64]. 

The cardiometabolic protective effects of plant-based ALA in humans are still unclear, as reported data on its influence on adiposity markers, blood pressure, dyslipidemia, and insulin insensitivity differ widely in studies [65,66,67,68,69,70]. Natural sources of nutrients are generally preferable in dietary treatments, and enough ALA is easily provided by their use. Since flaxseed and walnut have been frequently used as dietary sources of ALA, we briefly overview the effects of their consumption on obesity measures, dyslipidemia, glucose homeostasis, and inflammation markers in the next section. We summarize current knowledge in this scientific area considering recently published systematic reviews dealing with the effect of flaxseed and walnuts on cardiometabolic parameters and present them in Table 2. 

A meta-analysis of the available data regarding the effectiveness of interventions with walnuts on body weight, BMI, fat mass, and waist circumference was reported in the systematic review of Fang et al. [71], suggesting that walnut consumption does not alter anthropometric indices. According to the literature data, walnut consumption does not significantly improve biomarkers of blood glucose control but influences adiponectin and leptin levels [72,73]. However, walnut intake might lead to favorable changes in flow-mediated dilatation and LDL-cholesterol levels in subjects with impaired glucose homoeostasis [74]. Furthermore, the meta-analysis of Li et al. [75] shows that walnut consumption is not an efficient blood pressure lowering strategy. Finally, the effects of nut consumption on inflammation and endothelial function were reviewed in a systematic review and meta-analysis, and the authors did not find consistent evidence for effects of nut consumption on inflammation and suggest that further studies are needed to clarify effects of dietary intake of nuts on inflammation state in obesity [76]. 

Considering effects of flaxseed consumption, the meta-analysis of Yang et al. [77] revealed that flaxseed consumption induces favorable changes in total cholesterol, LDL-cholesterol, triglycerides apo-B and IL-6 but not apo-A, HDL-C, hs-CRP, CRP, or anthropometric indices in subjects with elevated blood pressure. The IL-6-lowering effect is attributed to flaxseed oil in this and another meta-analysis [77,78]. In addition, evidence shows that flaxseed can improve the HDL levels in healthy non-hyperlipidemic subjects and LDL only in a sub-group of overweight or obese ones [79]. In addition, the consumption of whole flaxseed may improve glycemic control and lower blood pressure slightly if it is consumed for more than 12 weeks [80]. According to the results of systematic reviews regarding the impacts of dietary intake of flaxseed, its nutritional use may be recommended for the prevention and management of obesity and obesity-associated features as presented in Table 2. However, it seems that favorable effects could not be attributed to ALA present in this seed but to other components present in flaxseed.

**Table 2:** Summary of findings of meta-analysis regarding the effects of flaxseed and walnuts on selected parameters.

Food Item	Reference	Study Population	Anthropometric Parameters	Blood Pressure	Serum Lipids	Insulin Sensitivity	Inflammation Parameters
Flaxseed	Yang et al., 2021 [77]	Dyslipidemic	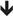 Body weight		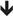 TC 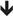 LDL-C 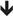 TG 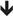 Apo-B 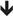 TC/HDL-C		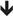 IL-6 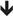 CRP
Flaxseed	Masjedi et al., 2021 [79]	Dyslipidemic Overweight			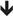 TC 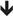 LDL-C 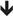 TG 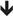 LDL-C		
Flaxseed	Khalesi et al., 2015 [80]	Metabolic syndrome, Type 2 diabetes, Hyperlipidemic, Peripheral artery disease		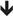 SBP 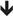 DBP			
Flaxseed oil	Yang et al., 2021 [77]	Dyslipidemic					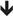 IL-6
							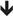 hs-CRP
Walnut	Yang et al., 2020 [72]	Metabolic syndrome,				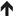 Leptin levels	
		Type 2 diabetes,				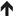 Adiponectin levels	
		Overweight with hypertension and/or hypercholesterolemia,				No changes in FG,	
		Healthy				HbA1c and insulin levels	
Walnut	Neale et al., 2020 [73]	Metabolic syndrome, Type 2 diabetes, Overweight				No changes in FG, HbA1c and insulin levels and HOMA-IR	
Walnut	Malmir et al., 2021 [74]	Abnormal glucose homoeostasis.	No changes in body weight and waist circumference	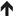 FMD No changes in SBP and DBP	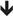 LDL-C	No changes in FG and HbA1c	
Walnut	Fang et al., 2020 [71]	Metabolic syndrome, Type 2 diabetes, Overweight and obese, Dyslipidemic, Cancer, Healthy	No changes in body weight, fat mass and waist circumference				
Walnut	Li et al., 2020 [75]	Metabolic syndrome, Chronic kidney disease, Diabetes or at high risk for diabetes, Moderate hypercholesterolemia, Healthy		No changes in SBP and DBP			

Abbreviations: Apo-B, apolipoprotein-B; CRP-C reactive protein; DBP, diastolic blood pressure; FG, fasting glucose; FMD, flow mediated dilatation; HbA1c: glycated hemoglobin; HDL, high-density lipoprotein; HOMA-IR, Homeostatic Model Assessment of Insulin Resistance; hs-CRP, high-sensitivity *CRP;* IL-6, interleukine 6; LDL, low-density lipoprotein; SBP, systolic blood pressure; TC, Total cholesterol; TG, triglycerides, 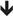 meaning that the parameter is shown to be decreased and 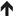 meaning that the parameter is shown to be increased after flaxseed or walnuts dietary intake.

## 6. New Findings on Mechanism of ALA Action In Vivo and Its Anti-Lipolytic Effect 

**Figure 2:**
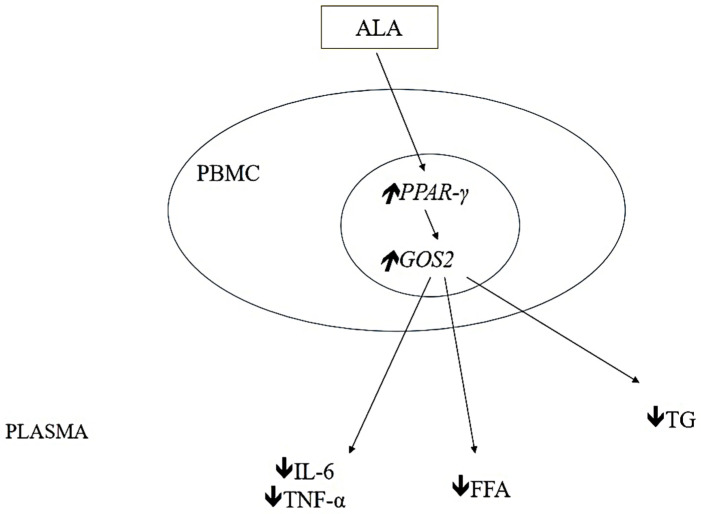
Anti-lipolytic mechanism of ALA. Abbreviations: *GOS2*, *GO/G1 switch gene 2*; FFA, free fatty acids; PBMC, peripheral blood mononuclear cell; *PPAR-γ, proliferator-activated receptor gamma*; IL-6, interleukin−6, TGs, triglycerides; TNF-α, tumor necrosis factor alpha, 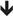 meaning that the expression or concentration of the parameter is reduced and 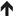 meaning that the expression or concentration of the parameter is increased after ALA treatment.

The beneficial effects of n−3 PUFAs in the prevention and treatment of obesity-related metabolic diseases such as type 2 diabetes, atherosclerotic cardiovascular disease, and nonalcoholic fatty liver disease may at least partly be attributed to their anti-lipolytic effect [81,82]. The GO/G1 switch gene 2 (GOS2) is a regulator of lipolysis and is a direct proliferator-activated receptor gamma (PPAR-γ) target gene [83]. At the same time, pharmacological studies indicate that PPAR-γ is a molecular target of n−3 PUFA [84,85]. In a recent study, Zhao et al. [81] tested the hypothesis that ALA elevates GOS2 expression and expresses an anti-lipolytic effect in peripheral blood mononuclear cells (PBMCs) of obese subjects. Indeed, after 12 weeks of treatment with about 4g of purified ALA plasma concentrations of TGs, FFA, glycerol, IL-6, and TNF-α were significantly lower in treated obese patients compared to the control group [81]. Furthermore, ALA increased PPAR-γ and G0S2 gene expression in PBMCs in parallel with the decrease in plasma FFA levels in obese patients, and changes in PPAR-γ and G0S2 gene expression in PBMCs correlated with the decrease in plasma FFA concentration, implying that G0S2 might mediate the anti-lipolytic effect of ALA [81] as presented in Figure 2. Finally, the findings of this study provide important data on the molecular mechanism of ALA’s anti-lipolytic action and thus possible therapeutic implications in obesity-related metabolic disorders.

## 7. A New Approach to Weight Control Using Diacylglycerol Oils Enriched in ALA

The potential of conventional edible ALA-rich oils (linseed and perilla) to improve fat metabolism and consequently reduce body fat has been reported in animal studies [86] but not in humans [87,88]. Triacylglycerols are major constituents in conventional edible oils, while diacylglycerol (DAG) and monoacylglycerol content is about 10%. Dietary consumption of DAGs that are not easily resynthesized to TAG leads to a reduction in postprandial lipid levels in humans compared to conventional TAG-rich oils [89,90]. Data from several studies in the last decade have shown that ALA-DAG consumption may be useful in the management and prevention of obesity without side effects [36,91,92]. In an intervention study in normal-weight and moderately obese men and women [36], a dietary intake of 2.5 g of ALA-DAG for 4 weeks increased dietary fat oxidation compared to ALA-TAG intake, leading to a significant decrease in the visceral fat area after ALA-DAG dietary treatment compared to effects found in the ALA-TAG control group. In two other studies, dietary daily consumption of equal amounts of 2.5 g/d ALA-TAG or ALA-DAG for 12 weeks in overweight [91] and obese subjects [92] led to a reduction in visceral fat area and BMI. In addition, plasma TG levels were reduced in the study with overweight participants [91] and WC in that with obese subjects [92]. In both studies, no ALA-DAG-associated adverse effects were detected in important urinary, hematologic, or blood biochemical parameters [91,92]. 

## 8. The Effects of ALA Consumption on Blood Pressure and Vascular Parameters in Obesity

The current knowledge of ALA effects on vascular and postprandial phases is understudied. At the same time, data on the potential effect on blood pressure are inconsistent [65,67,68,69,70]. In a recent placebo-controlled trial, high dietary consumption of ALA (4.7 g) for 12 weeks did not significantly affect 24 h ambulatory blood pressure or office blood pressure levels in men and women with (pre-) hypertension [93]. Furthermore, exploring the effect of the treatment with ALA on vascular function the authors revealed that using flaxseed oil in overweight and obese untreated (pre-) hypertensive individuals for 12 weeks compared to the high-oleic acid control group did not change brachial artery flow-mediated vasodilatation, carotid-to-femoral pulse wave velocity, retinal microvascular calibers, or plasma markers of microvascular endothelial function during the fasting and postprandial phase. Among all measured biomarkers, flaxseed oil dietary treatment significantly reduced fasting plasma FFA and TNF-α plasma contents. The ALA did not affect other markers of lipid and glucose metabolism or parameters of low-grade inflammation, and there were no changes in these parameters in the postprandial phase [94]. These findings could be of great importance, as the authors explored effects on broad spectra of biological markers in this randomized placebo-controlled trial listed in Table 3. Finally, the results of this investigation suggest that FFA and TNF-α could be key molecules involved in the expression of in vivo ALA action.

## 9. Effect of ALA on Obesity-Associated Non-Alcoholic Liver Disease

According to the literature data, n−3 PUFAs are involved in the regulation of lipid metabolism and could reduce ectopic fat accumulation in the liver [95,96,97,98]. Considering recent interventional studies with ALA in obesity, an isocaloric diet supplemented with 50g of refined rapeseed oil rich in ALA consumed for 8 weeks has shown a beneficial effect on intrahepatic lipid metabolism, as it induced a reduction in intrahepatic lipid content together with a significant decrease in serum FFA levels in obese men [99]. At the same time, according to the literature data, an energy-restricted diet could be the first-line treatment for NAFLD subjects [100,101]. A hypocaloric diet enriched with ALA-rich flaxseed oil induced favorable but similar changes compared to a control energy-reduced diet on fatty liver grade, liver enzymes, and cardiometabolic parameters [102,103]. The only significant difference noticed comparing these two energy-restricted diets was that the weight loss was higher in the group on a diet enriched with flaxseed [103]. The circulation and tissue status of PUFA were not estimated in the mentioned recent studies by Kruse et al. [99] and Santurino et al. [103] that investigated the effect of ALA-rich diets (isocaloric and hypocaloric, respectively), on lipid accumulation in the liver in obesity. At the same time, the monitoring of PUFA status during energy restriction treatment of NAFLD in obesity has been an important biomarker associated with the efficiency of dietary treatment. Although Ristic-Medic et al. did not evaluate the effects of ALA treatment on NAFLD parameters in obese patients, they have revealed that there is a significant connection between changes in n−3 PUFA status and parameters of lipid accumulation in obesity during calorie-restricted diets. Finally, the authors observed that participants on the Mediterranean diet had higher levels of DHA in serum phospholipids compared to subjects on the low-fat diet, indicating that n−3 PUFA status analysis could be of critical importance for clarifying the effects of n−3 PUFAs in NAFLD [104]. 

## 10. Comparison of Effects of ALA and DHA+EPA

ALA is present in high amounts in some plant-based oil, while the main dietary sources of EPA and DHA are seafood products [105]. To our knowledge, there is generally more literature evidence that confirms the beneficial effects of fish oil consumption on cardiometabolic parameters in obesity compared to those for ALA [106,107,108]. In several recent studies, the effects of ALA and EPA+DHA consumption on cardiometabolic parameters in obesity were compared [109,110,111].

The effects of marine- and plant-derived n−3 PUFA supplements on cardiometabolic profiles in middle-aged and elderly Chinese hypertensive patients with abdominal obesity have been studied by Yang et al. [109]. Participants consumed an enriched diet for 12 weeks: fish oil (FO, 2 g/day EPA+DHA), flaxseed oil (FLO, 2.5 g of ALA), and corn oil served as a control (CO). Blood pressure, lipid parameters, waist circumference, fasting glucose, and insulin were measured, and cardiometabolic risk scores were calculated. They were significantly lower in the FO group compared to the CO group after the intervention but not in the FLO group. This study indicates that marine n−3 FA intervention may improve cardiometabolic health and that it is more efficient compared to plant-based ALA. 

In the study of Baril-Gravel et al. [111], men and women with abdominal obesity and at least one other criterion for metabolic syndrome consumed five experimental isoenergetic diets for 4 weeks each, separated by 4-week washout periods. Each diet provided 60 g/3000 kcal of different oils: (1) control corn/safflower oil blend (CornSaff; LA-rich), (2) flax/safflower oil blend (FlaxSaff; ALA-rich), (3) conventional canola oil (Canola; OA-rich), (4) high oleic canola oil (CanolaOleic; highest OA content), (5) DHA-enriched high oleic canola oil (CanolaDHA; OA- and DHA-rich). Canola DHA oil was more efficient in increasing the expression of adiponectin and reducing the relative expression of IL 1B compared to the consumption of other oils. 

The obesity-associated inflammation effect of ALA supplementation has been understudied. Pauls et al. [110] compared the effect of 4g of ALA (in the form of flaxseed oil) or an equal dose of DHA (in the form of fish oil) on plasma oxylipins and plasma FA, inflammatory markers, and monocyte glucose metabolism as secondary outcomes of the study in women with obesity. The fish oil supplementation induced a significant increase in plasma levels of oxylipins derived from EPA and DHA, while there was no change in these oxylipins using flaxseed oil after 28 days of supplementation. ALA-rich flaxseed oil supplementation alters monocyte bioenergetics. The authors have shown that both ALA and DHA+EPA could exert unique effects on the immune system in obesity.

## 11. Effect of ALA Consumption on FA Composition in Obesity

Dietary ALA can be metabolized by elongation and desaturation enzymes to form EPA and DHA in humans [1]. However, the extent to which this bioconversion occurs as well as whether ALA exerts beneficial physiologic effects independent of its role as a precursor for EPA and DHA are not completely explored [1,6,7,8]. Further intervention studies with pure ALA determining fatty acids composition in circulation and tissues and monitoring changes in n−3 PUFA contents could contribute to answering these scientific questions. 

Consumption of ALA-rich diet in humans for several weeks to months led to an increase in ALA and/or EPA content in plasma/serum in numerous previous human trials [27,28,29,30]. Moreover, Silva et al. [31] reported in their systematic review regarding the impact of chia consumption on serum fatty acid profiles including n−3 PUFA that its dietary intake elevates ALA and EPA levels while decreasing DHA and showing no effect on DPA levels. The authors concluded that dietary intake of chia, being rich in ALA, has a neutral or potentially favorable effect on some markers of FA status, e.g., EPA [31]. Since changes in endogenous PUFA metabolism have been observed in numerous studies in obese subjects [22,39,40,41,42,43,44,45,46], we briefly summarize findings of two recent dietary interventional studies with ALA that monitored changes in FA profiles in plasma/serum in overweight/obese subjects during supplementation [112,113]. We have chosen these two studies, as, at first sight, the authors obtained completely different results regarding the impact of with ALA in obesity on FA composition and features associated with obesity. After supplementation with ALA, Egert et al. [113] found no change in ALA or EPA or decrease in DHA content accompanied by numerous favorable effects on cardiometabolic factors in overweight/obesity subjects with metabolic syndrome, while Nieman et al. [112] detected changes in plasma ALA and DHA content without an effect on parameters that can distinguish metabolically healthy and unhealthy individuals. 

**Figure 3:**
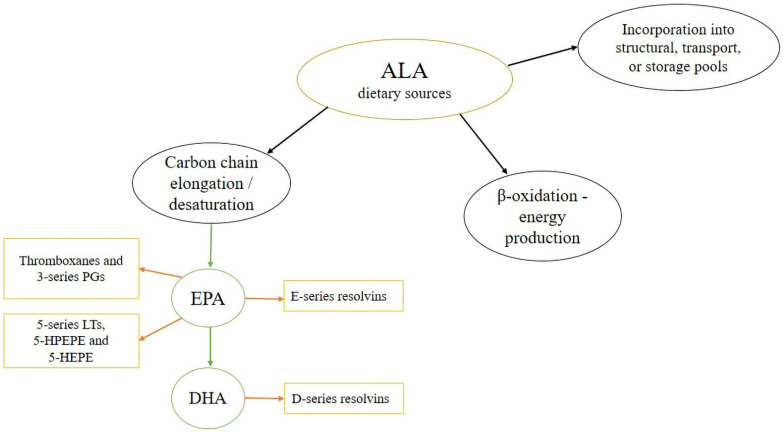
Overview of the metabolic fate of ALA. Abbreviations: ALA, alpha-linolenic acid; DHA, docosahexaenoic acid; EPA, eicosapentaenoic acid; HEPE, hydroxyeicosapentaenoic acid; HPEPE, hydroperoxyeicosapentaenoic acid; LT, leukotriene; PG, prostaglandin.

Egert et al. [114] found that an energy-restricted diet enriched with 3.7 g of ALA from rapeseed oil after 26 weeks did not systematically increase ALA or EPA, estimated by changes in PUFA status in serum phospholipids in overweight and moderately obese patients with metabolic syndrome. Furthermore, DHA levels decreased in both ALA-enriched and control diets. The results of this study that specifically investigated the metabolic fate of ALA during a hypo-energetic diet were somewhat unexpected, suggesting that a long-term catabolic state may impair n−3 PUFA status. This research group have reported that an energy-restricted diet enriched with ALA was accompanied by a significant decrease in body weight, systolic blood pressure, dyslipidemia, insulin sensitivity, inflammation, and endothelial function parameters. However, they have pointed out that it is not clear whether these additional findings can be attributed to increased dietary ALA in subjects’ diets due to obtained data for serum PUFA status. In addition, the possible metabolic fates of dietary ALA are shown in Figure 3, and the authors assumed that during a prolonged catabolic state ALA was mainly oxidized to yield the required energy [114].

On the contrary, ingestion of a non-caloric-restricted diet supplemented with 25 g of milled chia seed (4.4 g ALA) for 10 weeks in overweight premenopausal women significantly increased plasma ALA and EPA compared to whole chia seed and placebo consumption [112]. However, these changes in PUFA profiles were not associated with changes in glucose, cholesterol, CRP, blood pressure, LDL, HDL, TGs, or nine cytokines as obesity risk factors. Obtained results agree with some other studies that reported that the effects of ALA intake on blood pressure, serum lipids, glucose, insulin sensitivity, and markers of low-grade systematic inflammation have been scarce [94,113,115]. The obtained findings were similar to those found for chia supplementation in two other studies [116,117] as presented in Table 4. Since DHA levels were maintained at the same level during the study, its content could play a critical role in the improvement of features that are typically associated with obesity. Furthermore, it could be suggested that fish oil as a dietary source of EPA and DHA intake is more efficient in the treatment and management of chronic diseases [118]. Finally, the metabolic fate of ALA could be different in treatment with an isocaloric ALA-rich diet compared to a hypocaloric diet enriched with this n−3 PUFA.

## 12. Effect of Stearidonic Acid Consumption on FA Composition 

Several supplementation studies in humans have shown that the consumption of SDA-rich plant oils more efficiently elevates the content of EPA in the circulation (serum/plasma, erythrocytes, polymorphonuclear cells, and peripheral blood mononuclear cells) compared to a control/ALA-rich diet [119]. The intake of 1.2 g of SDA and consuming 10 g of echium oil per day for 6 weeks significantly increased the proportion of EPA and docosapentaenoic acid in RBC membranes in overweight and obese subjects. The proportion of DPA n−3 was also elevated, while DHA content remained unaffected [120]. These findings are in line with data obtained in previous studies [121,122,123,124], although the increase in EPA was not sufficient to increase the omega-3 index (representing the sum of EPA and DHA percentage in red blood cells) as has been reported in several studies [121,124]. The use of SCD in the diet of overweight/obese subjects could be beneficial regarding numerous EPA-induced beneficial health effects in obesity and related diseases [6]. In addition to the effect of SCD acid, the rate of bioconversion can be increased by sex hormones and zinc due to the upregulation of desaturase/elongase activities, which was also confirmed in studies by our research group [125,126,127,128]. Their potential to elevate the omega-3 index in obese subjects after treatment with ALA could be explored in further studies. 

## 13. Conclusions

In this narrative review, we briefly summarize current knowledge of:

1. *Changes in fatty acid profiles in obesity*

The endogenous metabolism of fatty acids induces changes in PUFA profiles in overweight and obese subjects. In addition to the almost uniform finding that delta-9 activity is increased, inducing changes in palmitoleic acid level, an increase in delta-6- and decrease in delta-5-desaturase activity involved in PUFA metabolism have also been observed. Considering available literature data these changes in desaturase activities are accompanied by an increase in DGLA amounts in plasma and other body compartments. In addition to these changes considering n−6 PUFAs, the elevated content of AA content is often found in adipose tissue.

2. *Effects of natural dietary sources (walnuts, flaxseed, and flaxseed oil) on features associated with obesity*

Obesity-associated persistent low-grade inflammation could be a primary target during intervention with n−3 PUFAs including ALA. The systematic reviews regarding effects of dietary consumption of flaxseeds and walnuts on weight suppression and common features observed in obesity including hypertension, characteristic dyslipidemia with high triglycerides, and low HDL-levels and insulin sensitivity have shown that among all these factors, flaxseed oil rich in ALA induces significant changes in marker of systematic inflammation. At the same time, according to literature data summarized in these systematic reviews, flaxseed supplementation representing a combination of high fiber, high lignan, and high ALA shows favorable changes including body weight, dyslipidemia, and blood pressure but no conclusive evidence for association with improvement of glucoregulation markers.

3. *Anti-lipolytic effect of ALA consumption*

It is well known that dietary intake of ALA acts in vivo through the induction of an anti-lipolytic effect. The G0/G1 switch gene in PBMCs of obese subjects was identified as a molecular target of ALA action in vivo leading to a decrease in TNF-α production and thus may represent a molecular link between ALA intake and decrease in inflammation. The obtained results also indicate that the ALA effects on expression G0/G1 are achieved through the PPAR-γ receptor. The effects on fasting FFA and TNF-α were also observed in a recent controlled trial in spite of the fact that the author did not show a connection with blood pressure, vascular function markers, or postprandial response of these markers.

4. *Role of ALA in NAFLD*

As obesity represents a health problem of excessive accumulation of fat primary in adipose tissue but also in ectopic localization, most intensively in the liver, an energy-reduced diet is the primary dietary strategy, and an ALA-rich diet could induce favorable changes compared to control energy-restricted ones.

5. *Potential of DAG oils enriched in ALA to reduce adiposity*

A research group explored the potential of lipids enriched in DAG-ALA compared to TAG-ALA regarding their impact on reduction in weight and adiposity markers, showing that the use of DAG oils is a promising new approach for effective reduction in adipose tissue mass.

6. *Comparison of ALA to EPA+DHA consumption effects in obesity*

Results of several studies that compared the effects of EPA+DHA to ALA supplementation generally confirmed that marine n−3PUFA has a more favorable impact on obesity markers and features associated with obesity that led to the progression of obesity to metabolic syndrome. In summary, the effect of several factors expressed as z-score may be more informative in the investigation of the roles of EPA+DHA and/or ALA showing a higher impact of EPA+DHA on cardiometabolic risk. However, the study shows that DHA and ALA consumption may have unique effects, DHA on EPA and DHA synthesis and ALA on bioenergetics in monocyte cells.

7. *Effects of ALA treatment on n−3PUFA profiles*

Two recent studies that monitored changes in PUFA profiles during supplementation with ALA were chosen to emphasize the importance of serum/plasma and tissue profile determination to outline current knowledge regarding ALA effects in vivo. In summary, these two trials show that ALA supplementation could induce changes in plasma/serum n−3PUFA profiles without beneficial effects on cardiometabolic risk factors, but without changes in plasma profiles favorable changes could be induced. At the same time, the potential of stearidonic acid to enhance endogenous synthesis of n−3PUFA shows that EPA was synthesized during supplementation, but the effect on omega-3 index was not observed in obesity. 

Future studies estimating the effects of ALA consumption on PUFA profiles in different body compartments could greatly expand current knowledge of ALA effects on weight suppression and factors that are associated with obesity. The need for molecular studies regarding lipid mediator status is also evident from a recent comprehensive study exploring oxylipin profiles in adipose tissue of obese subjects. At this moment, it seems that EPA+DHA induce more favorable changes in obesity compared to dietary treatment with ALA. However, ALA could exert a unique biological effect independent of its conversion to EPA and DHA, for example in bioenergetics of monocytes. At the same time, the anti-lipolytic effect that leads to a decrease in FFA could be a key mechanism for ALA’s in vivo action. The questions that arise at this moment are whether more efficient bioconversion of ALA is needed to enhance ALA effects and whether it can be achieved in obesity using dietary sources rich in stearidonic acid or some other factors that influence desaturase and/or elongase activity. Finally, the Mediterranean diet, being a combination of increased nuts and fish intake along with olive oil use as primary conventional edible fat, may represent a potent dietary pattern for prevention and management of obesity and obesity-associated features as it could lead to an increase in dietary intake of ALA together with EPA and DHA. 

## Figures and Tables

**Table 1:** ALA content in selected main dietary sources and its contribution to adequate intake.

	18:3n−3 (g/100g)	Source	Amount Needed to Meet Adequate Intake for Men (g or mL for Oils) *	Amount Needed to Meet Adequate Intake for Women (g or mL for Oils) *
**Seeds and legumes**				
Chia seed	17.83	Serbian Food Composition Database [32]	9.0	6.2
Flaxseed	17.09	Turkish Food Composition Database [33]	9.4	6.4
Rapeseed	2.46	Turkish Food Composition Database [33]	65.0	44.7
Soybean	1.29	Serbian Food Composition Database [33]	124.0	85.3
**Nut and kernel**				
Walnut	6.18	Turkish Food Composition Database [33]	25.9	17.8
Pecan	1.05	Turkish Food Composition Database [33]	152.4	104.8
**Fats and oils**				
Flaxseed oil	53.38	USDA Database [34]	3.0	2.1
Perilla oil	51.5	Lee et al., 2015 [35]	3.1	2.1
Soybean oil	6.78	Serbian Food Composition Database [32]	23.6	16.2
Canola oil	5.80	Turkish Food Composition Database [33]	27.6	19.0

* Adequate intake of ALA is 1.1 g/day for women and 1.6g/day for men [37].

**Table 3:** The list of parameres that were tested in Jorgis et al. study [94].

**Vascular function measures**
Carotid-to-femoral pulse wave velocity
Central augmentation index adjusted for heart rate
Central systolic blood pressure (SBP)
Central diastolic blood pressure (DBP)
Central retinal arteriolar equivalent (CRAE)
Central retinal venular equivalent (CRVE)
Retinal arteriolar-to-venular diameter ratio
**Microvascular endothelial function parameters**
Soluble vascular cell adhesion molecule (sVCAM)
Soluble intercellular adhesion molecule (sICAM)
Selectin
**Metabolic risk parameters**
Total cholesterol
HDL-cholesterol
LDL-cholesterol
Triglycerides
Free fatty acids (FFA)
Apolipoprotein A-1
Apolipoprotein B-100
Glucose
Insulin
HOMA index
**Low-grade systematic inflammation biomarkers**
Interleukin 6 (IL-6)
Interleukin 8 (IL-8)
Tumor necrosis factor α (TNF-α)
C-reactive protein (CRP)
Serum amyloid
Monocyte chemoattractant protein 1 (MCP-1)

Parameters that were tested in the study investigating effects of 12 weeks supplementation with 10 g/day of flaxseed by Joris et al. [94] in a randomized trial with untreated (pre-) hypertensive individuals.

**Table 4:** Summary of results from interventional studies regarding the effects of chia on selected parameters.

Reference	Study Design	Participants Characteristics (n)	Weeks	Daily Dose	Anthropometric Parameters	Blood Pressure	Serum Lipids	Insulin Sensitivity	Inflammation Parameters	Oxidative Stress Markers
Toscano et al., 2015 [116]	Randomized, placebo controlled	Overweight/obese men and women (n = 26)	12	35 g	No changes in body weight and waist circumference compared to placebo		No changes in LDL-C and TG	No change in FG		
Nieman et al., 2012 [112]	Randomized placebo controlled, double-blinded	Overweight, healthy, postmenopausal women (n = 62)	10	25 g	No change in body mass	No changes in SBP and AI	No change in TC	No change in FG	No changes in IL-6, IL-8, IL-10, TNF-α and CRP	
Nieman et al., 2009 [117]	Randomized placebo controlled, single-blinded	Overweight/obese men and women (n = 76)	12	50 g	No change in body mass	No change in SBP	No changes in TC, LDL-C and HDL-c	No change in FG	No changes in IL-6, MCP-1, TNF-α and CRP	No changes in TEAC and plasma nitrite

Abbreviations: AI, augmentation index; CRP-C reactive protein; FG, fasting glucose; HDL, high-density lipoprotein; IL-6, interleukin 6; IL-8, interleukin 8; IL-10, interleukin 10; LDL, low-density lipoprotein; MCP-1, monocyte chemoattractant protein 1; SBP, systolic blood pressure; TC, Total cholesterol; TEAC, trolox equivalent antioxidant capacity; TG, triglycerides, TNF-α, tumor necrosis factor alpha.

## Data Availability

Not applicable.
